# 

**Published:** 1857-03

**Authors:** 


### CONTENTS.

ORIGINAL ESSAYS AND COMMUNICATIONS.

Vascularity of Dentine. By J. Richardson.... 261
Models and Casts. By H. R. Smith.... 272
Digitalis.... 276
Functions of the Nervous System—The Cerebrum. By Prof. C. B. Chapman, M. D. 277
The Dental Profession and its appropriate work. By Geo. Watt.... 289
Excavators. By J. Taft.... 300
Dental Chemistry of the Mouth. By Geo. Watt.... 305
Professional Studies of the Dental Pupil. P. Aydelott, D. D.... 310

PROCEEDINGS OF SOCIETIES.

Thirteenth Annual Meeting of the Mississippi Valley Association.... 322
Discussions of the Mississippi Valley Association.... 329
Minutes of the Fifth Annual Meeting of the Ohio Dental College Association.... 346

Miscellaneous.... 353

Remarks of Dr. E. Collins—Tooth within a Tooth—Dental Periodicals—Organization of the St. Louis Dental Society—Dental Surgery in Europe.

Editorial.... 368

Dental Record Books—A Word with our Patrons—New Dental Colleges—Resignation and Nomination—Apologetic—French Teeth—Teeth—Dental Wreath—Dentistry and the Microscope.

